# A Multi-Pronged Evaluation of a Healthy Food Access Initiative in Central Texas: Study Design, Methods, and Baseline Findings of the FRESH-Austin Evaluation Study

**DOI:** 10.3390/ijerph182010834

**Published:** 2021-10-15

**Authors:** Kathryn M. Janda, Nalini Ranjit, Deborah Salvo, Aida Nielsen, Nika Akhavan, Martha Diaz, Pablo Lemoine, Joy Casnovsky, Alexandra van den Berg

**Affiliations:** 1 UTHealth School of Public Health, Austin, TX 78701, USA; Nalini.Ranjit@uth.tmc.edu (N.R.); Aida.Alibegovic@uth.tmc.edu (A.N.); Nika.Akhavan729@gmail.com (N.A.); martha.p.diazmarin@uth.tmc.edu (M.D.); Alexandra.E.VanDenBerg@uth.tmc.edu (A.v.d.B.); 2 Michael and Susan Dell Center for Healthy Living, Austin, TX 78701, USA; 3 Prevention Research Center, Brown School, Washington University in St. Louis, St. Louis, MO 63130, USA; dsalvo@wustl.edu; 4 Centro Nacional de Consultoría, Bogotá 110221, Colombia; plemoine@cnccol.com; 5 Sustainable Food Center, Austin, TX 78702, USA; joy@sustainablefoodcenter.org

**Keywords:** multi-level evaluation, agent-based modeling, food security and healthy food access intervention, community-specific research, baseline data

## Abstract

Food insecurity and limited healthy food access are complex public health issues and warrant multi-level evaluations. The purpose of this paper was to present the overall study design and baseline results of the multi-pronged evaluation of a healthy food access (i.e., Fresh for Less (FFL)) initiative in Central Texas. The 2018–2021 FRESH-Austin study was a natural experiment that utilized a cluster random sampling strategy to recruit three groups of participants (total *n* = 400): (1) customers at FFL assets, (2) residents that lived within 1.5 miles of an FFL asset, and (3) residents from a comparison community. Evaluation measures included annual cohort surveys, accelerometers and GPS devices, store-level audits, and built environment assessments. Data are being used to inform and validate an agent-based model (ABM) to predict food shopping and consumption behaviors. Sociodemographic factors and food shopping and consumption behaviors were similar across the three groups; however, customers recruited at FFL assets were lower income and had a higher prevalence of food insecurity. The baseline findings demonstrate the need for multi-level food access interventions, such as FFL, in low-income communities. In the future, ABM can be used as a cost-effective way to determine potential impacts of future large-scale food environment programs and policies.

## 1. Introduction

### 1.1. Food Insecurity and Food Access as Public Health Issues

Food insecurity is a condition defined by an individual’s limited or uncertain availability or ability to acquire safe and nutritious foods in socially acceptable ways [1]. Food insecurity is considered a public health concern because of its association with numerous health issues such as obesity and overweight, malnutrition, anemia, diabetes, hypertension, and other chronic conditions [2,3,4,5,6].

Food insecurity is also a marker of economic disparities. In the United States (US), food insecurity just prior to the COVID-19 pandemic affected 11.8% of all US households [7]. In some states and specific localities, the prevalence of food insecurity was substantially higher, and in Texas, 14% of families were estimated to be food insecure at the time [7], as were 16% of households in the Greater Austin area. Austin is one of the most economically vibrant and fastest growing urban centers in the country, and also a highly demographically diverse area, with 48.3% of its population being non-Hispanic white, 33.9% Hispanic, and 7.8% Black, and also has stark economic inequities [8]. These inequities are further evident by the high prevalence of food insecurity (16%) which is a clear reflection of economic disparities in the city [8].

While food insecurity is commonly understood to mean lack of availability of food, conceptually, food insecurity is more complex and comprises four different factors: availability, access, utilization, and stability over time [9]. Of these, geographic food access, which is defined as the ability of an individual to find food retail within their community, is the most researched and discussed construct of food insecurity [10]. Access consists of geographic and economic access to safe and culturally relevant foods [9]. Consequently, policies to reduce the prevalence of food insecurity within a community intervention, such as placement of grocery stores in communities with limited access to food, often exclusively focus on factors related to geographic food access [10,11,12,13,14]. Another strategy to reduce the prevalence of food insecurity with a community or policy intervention is by attempting to address economic food access through food assistance or produce incentive programs like Double Up Food Bucks [15,16,17,18].

### 1.2. Food Insecurity and Geographic and Economic Food Access Disparities

There are notable disparities and inequities in food insecurity and food access, specifically by race/ethnicity, income, and other social determinants of health. Historically, communities of color (areas that are predominantly a racial/ethnic minority, such as Latino/a, Black, and Asian) and lower income households are more likely to experience food insecurity in the United States than people who are non-Hispanic white and people who live in higher income households [19,20,21]. In addition, people of color and lower income communities are less likely to have proximal or affordable healthy food retail options in or near their homes and neighborhoods and, therefore, must travel farther to access food geographically and economically [22,23,24].

### 1.3. Literature Gaps

While there have been various interventions to improve economic and geographic food access among groups and communities’ that experience the aforementioned disparities [25,26,27,28], the literature on comprehensive evaluations of these interventions or initiatives remains limited [25,26,29,30,31,32]. Most interventions to date have relied on a single strategy, such as a healthy corner store model, and evaluations often only focus on more process data, such as the extent to which customers utilize the new assets, and do not include impact evaluation data on behavioral health outcomes. In addition, many previous studies have only used self-report data as opposed to objective data. Evidence suggests that single-level, single-component strategies for community-level health behavior change, such as dietary behaviors, have only limited effectiveness and reach and, therefore, have low potential for sustainable impact. For community-wide and long-term issues, such as food insecurity, multi-level, multi-sectoral, and multi-component interventions are the ones most likely to be effective [33]. Thus, there is a need for the development and implementation of interventions that include multiple strategies and for evaluations that are comprehensive, include objective data, and measure more than one outcome.

### 1.4. Creation of the Fresh for Less Initiative

Health disparities are evident in Central Texas, where Eastern Travis County has historically had a larger population of Black and Latino households, lower median household income, fewer healthy food retail opportunities, and a higher prevalence of food insecurity as compared with Western Travis County [34,35,36,37]. Due to the fact of these inequities in food insecurity and healthy food access in Eastern Travis County, the City of Austin created the *Fresh for Less* (FFL) program [38]. Informed by formative qualitative work with key community stakeholders and successful strategies published in the literature [25,26,29,30,31,32], FFL was designed to improve geographic and economic access to healthy foods in historically underserved areas. This was accomplished through a cross-sector partnership enabling the strategic placement of non-traditional food retail outlets (farm stands, mobile markets, and healthy corner stores) that offered subsidized healthy food products and accepted food assistance benefits in prioritized zip codes (Eastern Crescent of Travis County) that had a high prevalence of obesity, high prevalence of chronic disease, and historically limited geographic access to healthy food retail. Food offerings at the farm stands, mobile markets, and healthy corner stores were informed by the City of Austin initiated formative qualitative work with key community stakeholders. However, fresh produce offerings at the farm stands and mobile markets were also limited by what could be grown in the Central Texas region (where the study was conducted).

FFL was first piloted in 2016 and utilized a multi-pronged strategy of three different types of non-traditional food retail outlets: farm stands, mobile markets, and healthy corner stores each operated by three different local non-profit organizations, and the City of Austin oversaw the implementation of the three programs. Farm stands were operated by a local non-profit organization Sustainable Food Center (SFC) with the assistance of members of the community. Farm stands sold exclusively fresh, locally, and organically grown produce at subsidized prices, accepted Supplemental Nutrition Assistance Program (SNAP, a food assistance program for low-income individuals and households, also formally known as Food Stamps), and accepted a SNAP incentive program called SFC Double Dollars. In 2018, there were five farm stands participating in FFL that were strategically placed in the prioritized zip codes at schools, recreation centers, affordable housing complexes, and libraries during high-traffic times.

Mobile markets were operated by a local non-profit organization Farmshare Austin. Mobile markets sold fresh, locally, and organically grown produce (similar to the farm stands), but they also sold organic staple goods, such as pasta, pasta sauce, cooking oils, honey, eggs, etc., at subsidized prices, accepted SNAP, and also accepted a SNAP incentive program called SFC Double Dollars (similar to Double Up Food Bucks) [15,18]. In 2018, there were nine mobile markets participating in FFL, and they were located in prioritized zip codes at schools, recreation centers, libraries, and churches.

Healthy corner stores were supported by a local non-profit Go! Austin/Vamos! Austin and utilized existing corner stores in prioritized zip codes that wished to participate in the program by agreeing to carry additional healthy products (as defined by the Food Trust [39]), such as fresh and canned fruits and vegetables, whole grains, and other products, and accepted SNAP and SFC Double Dollars. In 2018, there were eight health corner stores participating in FFL. Thus, as of fall 2018, there were a total of 22 participating FFL assets located throughout the Eastern Crescent of the greater Austin area: five farm stands, nine mobile markets, and eight healthy corner stores.

In April 2018, Foundation for Food & Agriculture Research (FFAR) provided funding to SFC and UTHealth School of Public Health to conduct a full-scale evaluation study, called the Food Retail: Evaluation Strategies for a Healthy Austin or the FRESH-Austin study, to assess the FFL Initiative. Although the FFL Initiative was already under way at the start of the evaluation, community awareness of the new/enhanced food retail outlets was still lacking. The overall goals of the multi-pronged and community-centered FFL evaluation was designed (1) to measure the impact of the FFL intervention of healthy food purchasing and consumption on community residents and (2) to develop and validate an agent-based model to predict changes in healthy food purchasing and consumption among the study’s population given different simulated policy expansion scenarios. The purpose of this paper was to describe the design and methods of the FRESH-Austin evaluation study and to present initial baseline data describing the study’s sample.

## 2. Materials and Methods

### 2.1. Study Overview

**Figure 1:**
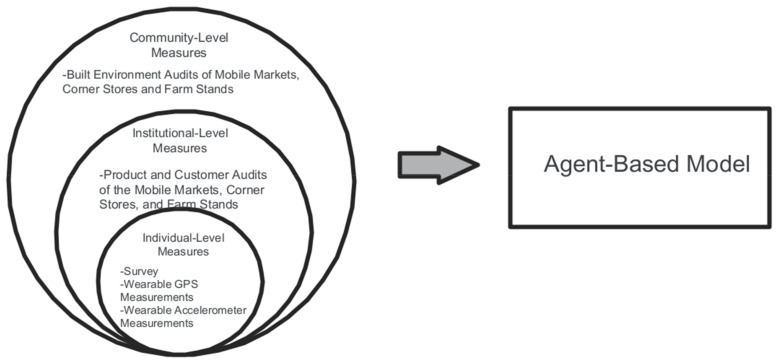
Levels of measurement of the FRESH-Austin study.

FRESH-Austin was a natural experiment consisting of a multi-level and multi-component, 3 year evaluation of the FFL Initiative. The overarching goal of the FRESH-Austin evaluation study was to understand if and how inter-related and inter-dependent food access interventions lead to sustainable solutions that promote health and increase economic opportunities, namely, by improving healthy food purchasing and intake behaviors. Given the multi-pronged nature of the intervention, the evaluation included empirical data on healthy food purchasing and consumption and of their potential determinants at the individual, institutional (food retail), and community levels. Because food purchasing and consumption behaviors are influenced by multiple factors and levels and given the multi-pronged nature of the interventions being assessed, we also developed and validated an agent-based model (ABM) (a complex systems simulation method) which was informed by the primary data collected in this study, and by pre-existing secondary data sources (see Figure 1). ABMs are incredibly helpful and useful when for exploring complicated, inter-dependent, and often expensive policy scenarios and their potential impacts on human behavior [33]. For this study, an ABM is being developed based on the various levels of data measurement presented in Figure 1. The resulting ABM will then be used to help model scenarios and policies designed to influence the food environment and resident behaviors in the greater Austin area. The FRESH-Austin study was approved by the University of Texas Health Science Center at Houston Institutional Review Board (HSC-SPH-18-0233).

### 2.2. Sampling and Recruitment

**Figure 2:**
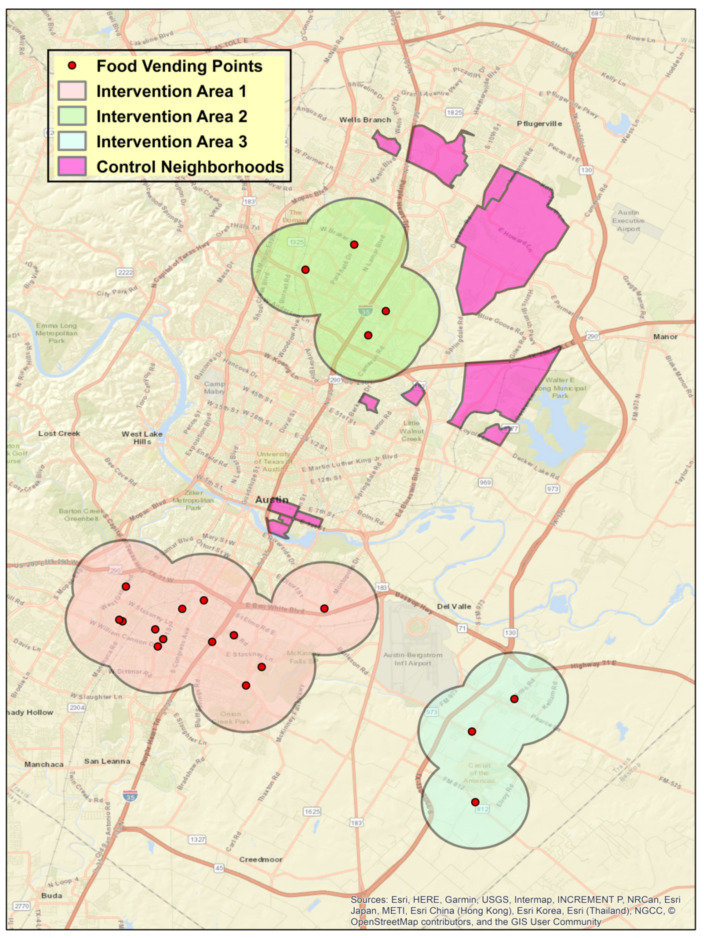
Map of FRESH-Austin Sampling Frame: The above presents the map of the sampling frame for the FRESH-Austin Evaluation study. The red dots are the locations of Fresh For Less assets that were open in 2018 and where the Confirmed Users were recruited. Intervention Areas (1, 2, and 3) are the areas where the Geographically Exposed participants were recruited, as they lived within 1.5 miles (or a 1.5 mile buffer) of an FFL asset. The control neighborhoods are where the Comparison Group participants were recruited, as these census tracts had similar sociodemographic characteristics as the census tracts of the geographically exposed areas, but they did not have an FFL asset within 1.5 miles of their community.

The FRESH-Austin evaluation studied collected primary data from 400 adult participants. Given the community-level nature of the study, we utilized a multi-stage cluster random sampling strategy, with three independent samples drawn from three groups: confirmed users of an FFL asset (Confirmed Users Group, *n* = 130, with FFL assets defined as FFL farm stands, FFL mobile markets, and FFL corner stores); community residents who lived within 1.5 miles of an FFL asset (Geographically Exposed Group, *n* = 185); community residents who lived in neighborhoods with similar sociodemographic characteristics to the geographically exposed neighborhood (as per the 2017 American Community Survey data [36]) but did not have an FFL asset (Comparison Group, *n* = 85). This sampling frame is shown in Figure 2.

Among the Confirmed Users group, participants were recruited at each FFL asset after their purchase. For the Geographically Exposed and Comparison groups, FRESH-Evaluation data collection staff conducted door-to-door recruitment in randomly selected street segments within each area to achieve an adequate spatial distribution of participants from these geographically defined groups. Recruitment and baseline data collection took place between October 2018 and March 2019. Eligibility criteria for participation in the study included being the primary shopper for groceries in the household, speaking English or Spanish, being over the age of 18 years, and not planning to immediately move out of the greater Austin area. Exclusion criteria were having a medical condition that would prohibit them from consuming fresh produce. All participants provided written informed consent upon enrollment in the study and received US 25 in cash upon completion of the survey to compensate them for their time.

### 2.3. Overall Study Measures

The FRESH-Austin Evaluation study was designed to measure the impacts of the FFL program at various levels; these included measures at the individual, institutional, and community levels. A description of the measures are presented in Table 1. At the *individual level*, instruments included annual surveys with participants recruited into the cohort study as well as wearable GPS and accelerometer device measurements among a subsample (*n* = 100) of those who were recruited to complete the survey. At the *institutional* or store level, this included audits of the stores as well as store counts of customers during a standardized period of time. At the *community level*, built environment audits were conducted. 

#### 2.3.1. Cohort Survey

The cohort survey included scales/items measuring individual-level behaviors, such as fruit and vegetable consumption [43], purchasing, perceptions of the built environment [44], etc., as well as questions about sociodemographic characteristics such as race/ethnicity, household size, utilization of food assistance services, food insecurity [45], and other factors. Findings from the baseline cohort survey are presented in this paper, and findings from other components of the FRESH Evaluation study, including a longitudinal analysis of survey data across the three-year assessment period, will be presented in subsequent papers.

The FRESH-Austin baseline cohort survey was conducted between October 2018–March 2019 and was interviewer administered in the preferred language (English or Spanish) of the participant. The survey included self-reported sociodemographic information for age, gender, household size, race/ethnicity, food assistance utilization in the past 12 months, 2017 gross income, education level, language spoken at home, and other factors. Food insecurity was measured using the validated 2-item screener [45]. The screener includes the following two questions: “For the past 12 months… (1) We (I) worried whether our food would run out before we (I) got money to buy more, and (2) the food that we (I) bought just didn’t last and we (I) didn’t have money to get more” [45]. Answer options for both questions were “Often True”, “Sometimes True”, and “Never True”. Based on their answers, participants were categorized as “Never or Almost Never” or “Sometimes or Always” experiencing food insecurity over the last 12 months as per the screeners validation and scoring guidelines [45].

The main behavioral factors of interest were fruit and vegetable consumption, fruit and vegetable purchasing, and grocery shopping behaviors. Fruit and vegetable consumption was measured using a modified block food frequency questionnaire that was adapted for another study among SNAP recipients in Central Texas with a predominantly Hispanic sample [46]. The fruits and vegetables included in the modified block FFQ were apples, citrus, bananas, berries, grapes, melon, lettuce, dark leafy greens, broccoli or cauliflower, carrots, tomatoes, avocadoes, sweet potatoes, potatoes (not sweet), cabbage, peppers, corn, zucchini or other squash, and onions, and respondents could list up to four additional fruits and vegetables consumed outside of this list. For each fruit and vegetable, respondents were asked how many times a week or month they ate that fruit/vegetable and if they ate it, how much did they usually eat (either in pieces or cups). These quantities were then standardized into cups. Fruit and vegetable consumption was then aggregated to ascertain total fruit, total vegetable, and total fruit and vegetable consumption in cups per day. This modified food frequency questionnaire, titled the FRESH FFQ, was subsequently validated using 24 h dietary recalls [43]. The validation study found acceptable levels of agreement between the FRESH FFQ and 24 h dietary recalls, thus validating this instrument for measuring fruit and vegetable consumption among the FRESH sample [43].

Fresh fruit and vegetable purchasing was self-reported and utilized the same list of fruit and vegetables as the validated FRESH FFQ [43]. Participants were asked to report the amount of fruit and vegetables purchased by the number of items or the number of pounds. When participants reported the purchase in number of items, this number was converted to pounds using a standardized protocol for each fruit and vegetable in the aforementioned list. These individual pounds of fruits and vegetables were aggregated and resulted in the value of total pounds of fresh fruit, fresh vegetables, and total fresh fruit and vegetables. These values were then standardized to account for household size by developing total fruit, total vegetable, and total fruit and vegetables purchased by the household in pounds per capita per week.

Food shopping behaviors were assessed with individual self-reported items measuring the various types of food retail the household shopped at (i.e., supermarkets/large grocery stores, smaller grocery stores, convenience stores, etc.), how often they shopped for food (number of times a month), and if they reported using any of the FFL assets. Food shopping motivations were also assessed based on the primary motivation for choosing the store at which they shop. Participants were asked to rank a list of factors from most important to least important when deciding where to shop. These factors included quality of food, variety, quality of store, cost, and cultural variety. These answer options were informed by our previous work with this community and the literature [32].

#### 2.3.2. Device-Based Geolocation and Physical Activity Data

We used Geopositioning Systems (GPS, QStarz BT-1000XT) and accelerometer (Actigraph wGT3X-BT) monitors to collect objective data on spatial and movement patterns as they relate to the food environment among a subsample of study participants (*n* = 100, distributed evenly across the main study groups: 33 from the Geographically Exposed group, 33 from the Comparison group, and 34 from the Confirmed User group). Both devices were worn for a 7 day period (starting the day after they completed the cohort survey) during waking hours and were initialized to start collecting data simultaneously (i.e., data across devices were time-matched). Time-matched GPS and accelerometry data represent a powerful tool for understanding spatial-based behaviors such as traveling in a city for purchasing food or for commuting or participating in physical activity. Together, these devices provide data that will be used to objectively confirm visits to different food outlet points throughout the city, understand travel patterns for food purchasing and their variations by individual characteristics, and categorize different travel modes (including those that contribute to healthy living such as active travel by walking or cycling).

#### 2.3.3. Store Audits

We also collected audit/direct observation data for characterizing the *institutional (food retail/store)* environment where FFL stores are located using adapted versions of the Nutrition Environment Measures Survey—Corner Store (NEMS-CS) [40], and Farmers’ Market Audit Tool (F-MAT) [41]. For corner stores, the audit tool included questions about the presence of signage, cleanliness, organization of the store, parking, whether food assistance was accepted, and the number, variety, quality, and prices of fruit, vegetables, and other healthy foods sold the day of the audit. For farm stands and mobile markets, the audit tools included questions about the presence of signage, parking, visibility from the street, types of food assistance accepted, and number, variety, quality, and prices of fruits and vegetables sold the day of audit, and staple goods (if applicable).

#### 2.3.4. FFL Store/Market Counts

In addition, we conducted direct observation using standardized momentary time sampling to assess how many people visited each FFL asset during a 15 min period on pre-selected days. This time frame was selected in order to obtain a standardized comparison across different strategies (given that different strategies had different operating hours). All corner stores were assessed on weekdays, while farm stands and mobile markets were assessed on days that they were open. Trained observers recorded how many people entered the FFL asset within the pre-defined data collection period and further coded if each visitor purchased (a) any food item and (b) fresh fruits and vegetables during their visit.

#### 2.3.5. Built Environment Audits

At the neighborhood level, we used the abbreviated version of the MAPS tool to assess the built environment in the surrounding area (street-level) of each FFL asset [42]. The MAPS tool collects data to determine if street segments were sufficiently walkable, including information on destinations and land-use (including public transit stops), streetscape characteristics, aesthetics and social elements, and crossings and intersections [42]. This type of information is critical for understanding if the environments where new healthy food access points are placed are in fact easily accessible to the surrounding community. As such, this assessment is especially relevant for initiatives such as FFL, based on the principle of increasing geographic access to healthy food, as there is an assumption that people will shop in locations that are nearby (i.e., potentially reachable by walking or public transit, versus by car only). In this study, we collected MAPS data for the street segment and its corresponding intersections where each FFL asset was located (for a total of 20 street segments). Each segment was identified using the two crossings nearest to the specific location of each outlet, evaluating both sides of the street. All audits were conducted for approximately a month period (10 August 2018–18 September 2018) and only during the hours of 7–10 a.m. or 6–8 p.m. for consistency purposes. These times were selected because they were high-traffic times for commuting as well as for the safety of data collectors, since these data were collected during the hot summer months of Central Texas, USA.

### 2.4. Analysis Plan

The overall analysis plan for the FRESH-Austin study included constructing a multi-level data set where participant-level data (survey and device-based data, merged by participant ID) were nested within sampling groups (Confirmed User Group, Geographically Exposed Group, and Comparison Group/Geographically Unexposed Group). Among the Confirmed User Group, institutional-level variables from the NEMS audit tool and street-level variables from the MAPS audit tool can be assigned to each participant based on the FFL asset where they were recruited. For Geographically Exposed participants, further GIS analysis will be conducted to determine: (a) institutional (NEMS) and (b) street-level (MAPS) characteristics of their nearest FFL asset, overall and by type (farm stand, mobile market, and corner store), and overall availability of FFL assets within a 5 min walking distance from their homes (using a buffer-based analysis). This multi-level data set will be used to run mixed-effects regression models to estimate the effect of participant, institutional (food asset), and environmental (street-level) characteristics on food purchasing and food consumption behaviors among this sample of adult participants from low-income, predominantly Hispanic communities in Central Texas. This future analysis will include two main approaches: (1) Longitudinal analysis comparing geographically exposed and comparison group—there are not many significant differences between these groups, as we selected the comparison areas because they were similar demographically. This approach will seek to examine the effect of geographic access (distance and density to and of stores). (2) Different comparison looking at what are the characteristics of those who use these FFL assets vs. those that do not, (i.e., confirmed users + geographically exposed users vs. non-users (geographically exposed non-users and comparison group). Within this analysis, we will be controlling for geographic access and plan to examine what factors help explain who uses and who does not use these assets. Specifically, for both analyses we will use analyses such as multivariable and mixed-effects models so we can adjust for both individual and area-level differences in sociodemographic factors across groups. Additionally, depending on future research questions, we will consider other methods that will help control for the fact that this was a natural experiment and not a randomized trial, such as propensity score matching.

### 2.5. Simulating FFL Policy Expansion Scenarios and Their Impacts on Food Insecurity

The individual, institutional, and environmental primary data gathered in this study combined with data from secondary data sources, including area-level sociodemographic and health-related characteristics (e.g., from the American Community Survey and 500 Cities Project), are being used to inform the development of an agent-based model (ABM).

ABMs represent a powerful tool for examining complex, inter-dependent, and often expensive policy scenarios and their potential impacts on human behavior [33,47,48,49]. For this study, an ABM is being developed whereby the simulated baseline environment represents the City of Austin in terms of area-level socioeconomic distribution and in which the geographic placement of traditional food access points (e.g., supermarkets) as well as novel ones (i.e., those included in the FFL initiative) match reality at baseline per our evaluation timeline. The development of the baseline environment for the ABM was informed by Food Retail Access Point locational data from the City of Austin Food Environment Analysis [35]. 

Next, the simulated city was populated by “agents”, representing people, that were assigned a weekly budget for food depending on their income level. The way in which these simulated people (agents) decide on where, what type, and how much food they purchase were informed both by the primary empirical data collected as part of this study (Figure 1) as well as by secondary data sources including reports by the City of Austin, the state of Texas, and other research studies. A set of evidence-informed rules to define how food-purchasing and consumption decisions are made were developed, tested, and calibrated against real data. Next, a set of simulated policy expansion scenarios will be tested (e.g., higher density of a certain type of food store, cheaper prices, varying placement options, combinations of different food access interventions). This will allow for examination of the expected impacts of scaling up different combinations of food access interventions.

This systems-based approach represents a cost-effective way of understanding the potential impact of rolling out large-scale food environment policies that are often expensive and/or controversial and that are unlikely to receive sufficient support without prior evidence of their potential for success. Notably, this method does so in a way that not only accounts for but also embraces the complexity of human behavior and multi-pronged and multi-level interventions. The development of this model is currently underway. Potential scenarios to be tested with the ABM include expansion of FFL programming, pivoting FFL implementation strategies, effects on the local food system during times of crisis (e.g., COVID-19 pandemic, Winter Storm Urie (which occurred in February 2021 and greatly impacted food access and availability in the region), and other events that could affect food availability, geographic and economic food access, and individual mobility), policy changes to expand economic and geographic food access, and other scenarios.

### 2.6. Baseline Descriptive Data Analysis

In this paper, we present initial baseline descriptive findings based on survey data. Analytic methods used primarily consisted of simple two-way tabulations for categorical variables and summary statistics for continuous variables of interest across treatment conditions, without adjustment for other possible confounders. Chi-squared tests were utilized to examine differences in the distribution of levels of the categorical variables and ANOVA tests for continuous variables and outcome variables across the three groups of participants: Confirmed Users, Geographically Exposed, and Comparison area participants. *p*-Values from chi-squared analyses for categorical variables and F values, degrees of freedom, and *p*-values from ANOVA analyses for continuous variables are presented to assess the extent to which there were differences among the three groups. A threshold value of 0.05 was utilized to conclude if there were statistically significant differences.

Due to the sample size, some categories of race/ethnicity and other sociodemographic factors were collapsed. In addition, outliers were removed for the fruit and vegetable consumption data. All data analyses were conducted after extensive data cleaning and all statistical analyses were performed using Stata (version 16, College Station, TX, USA), and resulting graphs were created using R (R Team) and maps using ESRI Inc. ArcMap for geospatial analysis (version 10.6.1, Redlands, CA: Environmental Systems Research Institute).

## 3. Results

### 3.1. Sociodemographic Characteristics of the Cohort

The final sample included 400 participants who consented to be part of the cohort study and completed the baseline cohort survey. The descriptive statistics and results from chi-squared and ANOVA tests of the demographic characteristics of the sample stratified by recruitment category are presented in Table 2. Of the total sample, 130 participants were Confirmed Users, 185 were Geographically Exposed, and 85 resided in Comparison areas. Among the 130 Confirmed Users, 33 were recruited from participating healthy corner stores, 49 were recruited from mobile markets, and 48 were recruited from farm stands. The sample was predominantly female, identified as Hispanic/Latino, with the mean age being 44 years of age with no significant differences across groups. Participants predominantly spoke English at home, with no statistically significant differences among the groups.

However, there were significant differences between groups by income, education, food assistance utilization, and food insecurity status. For income, there were significant differences (*p* < 0.01) with Confirmed Users having a higher proportion of income under $45,000 and Geographically Exposed and Comparison participants having a higher proportion of income over $65,000. For education level, Confirmed Users had a higher proportion of less than a high school education while Geographically Exposed and Comparison participants had a higher proportion of having a college diploma or more (*p* = 0.04). Participants reported utilization of food banks (*p* = 0.02), free and reduced lunch (*p* < 0.01), and SNAP (*p* < 0.01) in the last year were all statistically significant across the three groups, with Confirmed Users having a higher proportion of utilization. Additionally, there were significant differences in food insecurity across the three groups (*p* < 0.01), with Confirmed Users having a higher proportion of food insecurity than Geographically Exposed or Comparison participants.

### 3.2. Fruit and Vegetable Consumption, Purchasing, Shopping Behaviors, and Motivations

**Figure 3:**
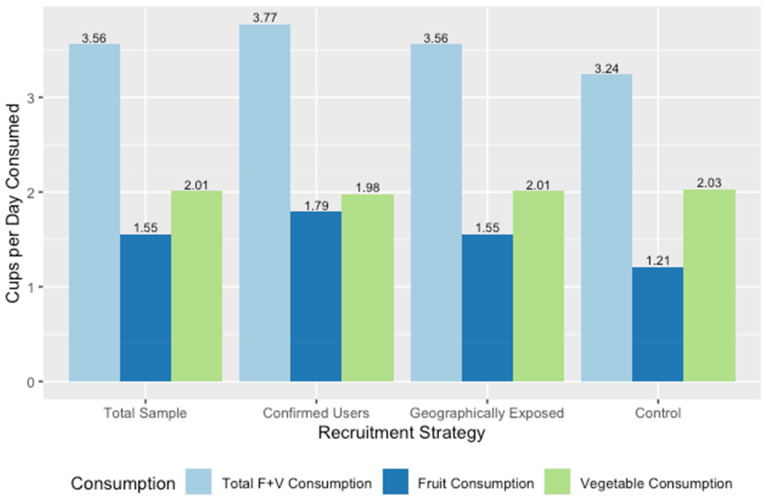
Average fruit and vegetable consumption in cups/day among adults at baseline by recruitment strategy (FRESH-Austin Evaluation study Austin, Texas, October 2018–March 2019).

Participants provided information about their fruit and vegetable consumption behaviors presented in Figure 3. Across all three groups there was greater reported vegetable (shown in green in Figure 3) consumption than fruit consumption (shown in dark blue in Figure 3). There were no significant differences in total fruit and vegetable consumption and vegetable consumption across the three recruitment groups. However, Confirmed Users reported significantly higher fruit consumption than Geographically Exposed or Comparison participants (F(2,2) = 8.70, *p* < 0.01).

Descriptive statistics and results from chi-squared and ANOVA tests for fruit and vegetable purchasing behaviors, shopping behaviors, and shopping motivations stratified by recruitment category are presented in Table 3. There were no statistically significant differences by recruitment group for fruit and vegetable purchasing (combined and fruit and vegetables separately), frequency of food shopping, and the most important factor for deciding where to shop for food. Similar to fruit and vegetable consumption, participants across all three groups reported buying more vegetables than fruit per week. In addition, across all groups, respondents reported that they either shopped for groceries weekly or more than weekly and that their number one factor for deciding where to shop was “quality of food” with no significant differences across groups.

However, there were some significantly different findings for types of stores shopped at. The overwhelming majority of the sample reported shopping at supermarkets (>98% for all groups). There were no significant differences by group for shopping at smaller grocery stores, convenience stores, or farmers’ markets. However, there were significant differences by recruitment strategy for shopping at mobile markets (*p* < 0.01) and farm stands (*p* < 0.01), with Confirmed Users having a higher proportion of reporting that they shopped at those assets than Geographically Exposed or Comparison participants. Additionally, there were significant differences among the three groups for the number of different types of food shopping locations (*p* < 0.01), with the majority of Confirmed Users reporting shopping at supermarkets and two other types of food retail, while the majority of Geographically Exposed and Comparison participants reported shopping at supermarkets and only one other type of food retail. Finally, and expectedly given that this group was recruited at one of the three FFL assets, Confirmed Users were significantly more likely to report shopping at all FFL assets including healthy corner stores (*p* < 0.01), mobile markets (*p* < 0.01), and farm stands (*p* < 0.01) than Geographically Exposed or Comparison participants.

## 4. Discussion

### 4.1. Summary of Study Overview

In summary, the purpose of the FRESH-Austin study was to conduct a multi-prong evaluation of the FFL Initiative, a multi-level food access intervention in Central Texas. Using a comprehensive approach, the FRESH-Austin study will examine the impact of the FFL Initiative at the individual and community levels through cohort surveys, audits, etc., which subsequently is informing the development and validation of an agent-based model.

This study is innovative in several ways, including the cluster randomized sampling strategy, multi-level evaluation design, and the use of agent-based modeling. The sample for the cohort included participants who were purposefully sampled: Confirmed Users of FFL, Geographically Exposed participants to FFL, and participants living in Comparison areas that had similar sociodemographic characteristics to Geographically Exposed areas but did not have an FFL present. This sampling approach and multi-level nature of the evaluation lends itself to measuring the individual and community-wide impacts of the FFL initiative, which often is not possible or feasible in interventions designed to mitigate food insecurity and increase access to healthy foods. To the best of our knowledge, a rigorous evaluation study of urban initiatives to improve geographic access to healthy food among low-income groups that combines survey, qualitative, device-based, and simulation modeling methods, has not taken place prior to ours. Furthermore, most food access interventions only employ one strategy, such as only offering healthy corner stores or mobile markets, and often do not have evaluation plans that assess more than one strategy [25,26,27,29]. Hence, our main contribution to the literature lies in the description of our unique methodological approach to assess the impact of the multi-pronged Fresh For Less initiative on healthy food purchasing and consumption among low-income communities within the context of a natural experiment.

In addition, the utilization of agent-based modeling is a cost-effective approach for understanding the potential impact of rolling out large-scale food environmental changes and policies [33,47,48,49]. For example, it is cheaper and faster (i.e., more cost-effective) to run a computational simulation of the placement of new supermarkets in low-income areas of a city, and to use the simulation results for examining potential changes in food purchasing and intake patterns than to actually open new physical stores and wait several years to measure their possible effect on food purchasing and intake behaviors. In fact, other similar modeling approaches for health behaviors (e.g., physical activity) have shown that the timeframe needed to observe population-level changes in health behaviors due to the fact of large-scale built environment transformations is often of five or more years. These types of timelines are much greater than typical research funding and political cycles. This is particularly important given that food environment interventions and policies can be controversial and are often expensive. Providing a stronger evidence base to support these types of programs or policies could result in greater likelihood for sufficient constituent support, paving the way for potential success. Agent-based modeling has tremendous potential for providing this much needed evidentiary support while also accounting for complex human behaviors and multi-pronged and multi-level interventions. As such, ABMs offer a powerful tool for using an evidence-informed approach to environmental modifications for reducing food insecurity and improving access to healthy foods. Adopting an evidence-informed approach exclusively based on empirical evidence would likely be impossible, making simulation methods a powerful tool for evidence-informed policy action. Next steps for this study include concluding the development and validation of this model.

### 4.2. Summary of Baseline Cohort Survey Findings

The findings presented in this manuscript were data obtained from the baseline cohort survey, which were collected between October 2018 and March 2019. Baseline results show that the three groups of participants were generally comparable; however, there were statistically significant differences among some sociodemographic characteristics and food consumption as well as shopping behaviors. Specifically, there were statistically significant differences by various sociodemographic factors including income, education, food assistance utilization, and food insecurity status. For the aforementioned factors, there were the most notable differences among Confirmed Users of FFL. This demonstrates that the FFL program is reaching its intended communities of interest, since they were designed to improve access to healthy foods among individuals with lower income and socioeconomic status and those experiencing food insecurity. Moreover, there were significant differences among various behavioral variables of interest including fruit consumption and shopping behaviors, specifically utilization of different types of food retail and FFL utilization. The significant differences in greater utilization of mobile markets, farm stands, FFL assets, and multiple types of food retail being driven by the confirmed user group is to be expected given the recruitment strategy. These differences in the sociodemographic and behavioral characteristics at baseline will be examined in future analyses and differences with subsequent years of the cohort survey.

Another interesting finding from the baseline results were higher fruit and vegetable consumption among the cohort. Specifically, fruit and vegetable consumption among the cohort was higher than the national average. This is hypothesized to be the case since the majority of the sample identified as Hispanic/Latino, and the literature has demonstrated that Hispanic/Latino Americans eat more fruit and vegetables than the average American [50]. However, future work should investigate this phenomenon further.

### 4.3. Strengths and Limitations

#### 4.3.1. Strengths

A notable strength to the FRESH-Austin study is the multi-pronged and multi-level nature of the evaluation. By measuring potential impacts of the FFL intervention at multiple levels, the evaluation can be sensitive to objective and subjective impacts at the individual, institutional, and community levels. This is needed in order to better capture the nuances required for such a complex issue as food insecurity and healthy food access. In addition, by utilizing a cluster randomize sampling strategy, the impact of the FFL program can be better examined between groups directly, indirectly, and not impacted by the program via multiple comparison groups. Moreover, by utilizing a cohort design, we are able to follow participants for three years, enabling us to better understand changes in fruit and vegetable consumption and purchasing, FFL utilization, healthy food access, food insecurity, and other factors over time.

#### 4.3.2. Limitations

However, there are limitations to this study. The FRESH-Austin study utilized a strategically selected sample, so while there was randomization, it may not be representative to the city or the Central Texas region. Moreover, there could be potential issues with sample size for the groups that wore accelerometers and GPS; this will be determined in future analyses. The dietary data in the cohort survey was measured using a food frequency questionnaire rather than 24 h dietary recalls; however, this measure was validated [43]. In terms of the baseline cohort survey analyses presented in this paper, only cross-sectional analyses and simple statistics are presented. Furthermore, the baseline survey was administered after FFL had started implementation but was the first year of formal evaluation. We recognize that the lack of pre-implementation data is certainly a limitation, but we obtained data from other studies that drew community samples from the same areas of the city [32], and our longitudinal assessment will allow us to assess trends and understand if the placement and utilization of the new food access points are making a difference in food purchasing/intake patterns, and will inform the ABM in running several expansion simulation scenarios. Future work will explore outcome-related data as well as more specific associations with additional rigorous analyses as described in the overall analytic plan in Section 2.

### 4.4. Public Health Implications and Next Steps

These findings demonstrate that the participants using the FFL were lower income and had a higher prevalence of food insecurity, demonstrating that those utilizing the program were the priority population. Additionally, future outcome work and agent-based modeling will help inform policymakers and stakeholders (non-profit service providers, government agencies, etc.) on the best strategies for tackling issues related to health, poverty, and food insecurity. This agent-based model can be built upon, allowing for additional layers of data not related to the food system directly (transportation, etc.) ensuring its use to a range of stakeholders.

## 5. Conclusions

In conclusion, this manuscript presents a summary of the FRESH-Austin study’s evaluation methods and also discusses the baseline cohort survey’s findings stratified by recruitment strategy. This is a valuable contribution to the literature given the lack of comprehensive, multi-level evaluations of food insecurity mitigation and healthy food access promotion programs in the literature and also outlines the role of innovative agent-based modeling. This approach is particularly relevant to modeling various scenarios, informed by community-specific data and has strong program and policy implications.

## Figures and Tables

**Table 1:** Table of the measures included in the FRESH-Austin study.

Instrument	Purpose	Type of Instrument	How Often Administered	How Administered
Cohort survey	To measure food purchasing, food consumption behaviors, food insecurity, and other factors	Self-report survey	Annually (October–March)	Administered by trained data collectors annually
Accelerometer	Objectively measure physical activity among a subsample	Device-based—Actigraph wGT3X-BT	Baseline (October 2018–March 2019)	Validated wear time for 7 days
GPS	Objectively measure interactions in the built environment	Device-based—GPS, QStarz BT-1000XT	Baseline (October 2018–March 2019)	Validated wear time for 7 days
Store audits	Measure the offerings available at the various types of FFL assets	Adapted versions of NEMS-CS ^1^ [40] and F-MAT ^2^ [41] for farm stands and mobile markets	Annually	Assessed by trained FRESH-Austin staff
Store counts	Count how many customers were at an FFL for a standardized period of time	Developed count tracker	Baseline (October 2018–March 2019)	Assessed by trained FRESH-Austin staff
Built environment audits	Measure aspects of the built environment in the immediate vicinity of FFL assets	Adapted version of MAPS [42]	Baseline (August 2018–September 2018)	Assessed by trained FRESH-Austin staff

^1^ Nutrition Environment Measures Survey—Corner Store; ^2^ Farmers’ Market Audit Tool.

**Table 2:** Descriptive statistics of sociodemographic characteristics stratified by recruitment strategy among adults at baseline (FRESH Evaluation study Austin, TX, USA, October 2018–March 2019).

Variable	Full Sample	Confirmed Users	Geographically Exposed	Comparison	*p*-Value/ANOVA
	*n* = 400	*n* = 130	*n* = 185	*n* = 85	
	% (N)/Mean (SD)	% (N)/Mean (SD)	% (N)/Mean (SD)	% (N)/Mean (SD)	
Gender					
Female	70.5% (282)	73.85% (96)	68.11% (126)	70.59% (60)	
Male	29.25% (117)	26.15% (34)	31.35% (58)	29.41% (25)	0.69
Age	43.89 [13.66]	43.45 [12.29]	44.35 [14.08]	43.56 [14.84]	F(2,2) = 0.19, *p* = 0.82
Race/Ethnicity					
Hispanic/Latino	54.41% (216)	60.77% (79)	54.10% (99)	45.24% (38)	
Black	10.08% (40)	6.15% (8)	10.38% (19)	15.48% (13)	
White/Other	35.52% (141)	33.08% (43)	35.52% (65)	39.29% (33)	0.12
Language Spoken At Home					
Spanish	6.75% (107)	31.54% (41)	28.11% (52)	16.47% (14)	
English	59.00% (236)	50.77% (66)	61.08% (113)	67.06% (57)	
Both Spanish/English	13.25% (53)	16.92% (22)	9.73% (18)	15.29% (13)	
Other	0.75% (3)	0.77% (1)	0.54% (1)	1.18% (1)	0.15
Household Size	3.47 [1.87]	3.54 [1.81]	3.61 [1.94]	3.06 [1.75]	F(2,2) = 3.04, *p* = 0.05
Household Income in 2017					
Under $25,000	23.04% (88)	32.81% (42)	16.09% (28)	22.50% (18)	
$25,001–$45,000	29.58% (113)	35.94% (46)	27.59% (48)	23.75% (19)	
$45,001–$65,000	18.32% (70)	17.19% (22)	18.97% (33)	18.75% (15)	
Over $65,000	29.06% (111)	14.06% (18)	37.36% (65)	35.00% (28)	**<0.01**
Education					
Less than High School	12.12% (48)	19.38% (25)	9.89% (18)	5.88% (5)	
High School or General Educational Development (GED) Graduate	21.72% (86)	24.81% (32)	20.33% (37)	20.00% (17)	
Some College	21.21% (84)	18.60% (24)	21.43% (39)	24.71% (21)	
College Graduate or More	44.95% (178)	37.21% (48)	48.35% (88)	49.41% (42)	**0.04**
Food Assistance *					
Utilized Food Bank in Last Year	12.00% (48)	18.46% (24)	9.73% (18)	7.06% (6)	**0.02**
Utilized Free and Reduced Lunch in Last Year	26.50% (106)	36.92% (48)	24.86% (46)	14.12% (12)	**<0.01**
Utilized SNAP in Last Year	17.50% (70)	26.92% (35)	13.51% (25)	11.76% (10)	**<0.01**
Utilized Women, Infants, and Children (WIC) in Last Year	9.25% (37)	13.08% (17)	8.11% (15)	5.88% (5)	0.16
Food Insecurity					
Sometimes or Often	39.60% (158)	49.61% (64)	37.30% (69)	29.41% (25)	
Never	60.40% (241)	50.39% (65)	62.70% (116)	70.59% (60)	**<0.01**

* Could select more than one answer option. Bold signifies significant differences at *p* < 0.05.

**Table 3:** Fruit and vegetable purchasing, food shopping behaviors and motivations among adults at baseline (FRESH Evaluation study Austin, Texas, (October 2018–March 2019).

Variable	Full Sample	Confirmed Users	Geographically Exposed	Comparison	*p*-Value/ANOVA
	*n* = 400	*n* = 130	*n* = 185	*n* = 85	
	% (N)/Mean (SD)	% (N)/Mean (SD)	% (N)/Mean (SD)	% (N)/Mean (SD)	
**Fresh Fruit and Vegetable Purchasing**					
F+V Pounds/Capita/Week	8.01 [6.25]	8.63 [7.16]	7.58 [5.94]	8.02 [5.33]	F(2,2) = 1.08, *p* = 0.34
Fruit Pounds/Capita/Week	3.36 [2.97]	3.59 [3.40]	3.26 [2.84]	3.22 [2.53]	F(2,2) = 0.59, *p* = 0.56
Vegtable Pounds/Capita/Week	4.65 [3.93]	5.03 [4.35]	4.32 [3.86]	4.80 [3.37]	F(2,2) = 1.34, *p* = 0.26
**Shopping Behaviors**					
Types of shopping locations (check all that apply)					
Supermarkets	99.25% (397)	99.23% (129)	99.46% (184)	98.82% (84)	0.85
Small Grocery Store	64.75% (259)	63.85% (83)	69.19% (128)	56.47% (48)	0.18
Convenience Store	22.25% (89)	27.69% (36)	19.46% (36)	20.00% (17)	0.41
Farmers’ Market	12.25% (49)	16.15% (21)	10.27% (19)	10.59% (9)	0.054
Mobile Market	15.25% (61)	41.54% (54)	3.78% (7)	0.00% (0)	**<0.01**
Farm Stand	13.00% (52)	38.46% (50)	1.08% (2)	0.00% (0)	**<0.01**
Other	1.51% (6)	0.00% (0)	2.70% (5)	1.19% (1)	0.15
Number of shopping locations					
Only Shopped at Supermarkets	17.50% (70)	6.15% (8)	20.00% (37)	29.41% (37)	
Supermarkets + 1 other type of store	45.00% (180)	25.38% (33)	55.14% (102)	52.94% (45)	
Supermarkets + 2 other types of stores	28.25% (113)	44.62% (58)	22.16% (41)	16.47% (14)	
Supermarkets + 3+ other types of stores	8.50% (34)	23.08% (30)	2.16% (4)	0.00% (0)	
Other Type of Stores	0.75% (3)	0.77% (1)	0.54% (1)	1.18% (1)	**<0.01**
Frequency of food shopping					
Shopped less than weekly	14.79% (59)	13.95% (18)	12.97% (24)	20.00% (17)	
Shopped weekly	42.36% (169)	38.76% (50)	46.49% (86)	38.82% (33)	
Shopped more than weekly	42.86% (171)	47.29% (61)	40.54% (75)	41.18% (35)	0.37
Shopped at Fresh For Less Location(s) *					
Shopped at Farm Stand	12.75% (51)	37.69% (49)	1.08% (2)	0.00% (0)	**<0.01**
Shopped at Mobile Market	15.00% (60)	40.77% (53)	3.78% (7)	0.00% (0)	**<0.01**
Shopped at Health Corner Store	5.00% (20)	12.31% (16)	2.16% (4)	0.00% (0)	**<0.01**
**Most Important Factor When Deciding Where to Shop for Food**					
Quality of Food	52.63% (210)	46.34% (57)	55.19% (101)	62.65% (52)	
Cost	25.96% (101)	27.64% (34)	24.04% (44)	27.71% (23)	
Variety of Food	12.34% (48)	15.45% (19)	13.11% (24)	6.02% (5)	
Quality of Store	4.88% (19)	4.88% (6)	5.46% (10)	3.61% (3)	
Cultural Variety	2.83% (11)	5.69% (7)	2.19% (4)	0.00% (0)	0.10

* Could select more than one answer option. Bold signifies significant differences at *p* < 0.05.

## Data Availability

Data can be shared by request upon contacting the authors.
